# Impaired DNAJB2 Response to Heat Shock in Fibroblasts from a Neuropathy Patient with *DNAJB2/HSJ1* Mutation: Cystamine as a Potential Therapeutic Intervention

**DOI:** 10.3390/neurolint17050073

**Published:** 2025-05-09

**Authors:** Raj Kumar Pradhan, Nikolas G. Kinney, Brigid K. Jensen, Hristelina Ilieva

**Affiliations:** Jefferson Weinberg ALS Center, Department of Neuroscience, Thomas Jefferson University, 900 Walnut Street, JHN 4th Floor Suite 400, Philadelphia, PA 19107, USA; rajkumar.pradhan@jefferson.edu (R.K.P.); nikolas.kinney@students.jefferson.edu (N.G.K.); brigid.jensen@jefferson.edu (B.K.J.)

**Keywords:** neuropathy, heat shock proteins, DNAJB2, HSP40, HSJ1, Cystamine

## Abstract

**Background and Objectives:** Neuropathy is a debilitating disorder characterized by peripheral nerve dysfunction and damage to sensory, motor, and autonomic neurons and their axons. While homozygous mutations in DNAJB2/HSJ1 have been linked to early-onset neuropathy, a heterozygous DNAJB2 c.823+6C>T was discovered in an adult patient with severe sensory–motor polyneuropathy. This mutation is predicted to affect both isoforms of the protein. DNAJB2 (HSP40), a key member of the heat shock protein family, plays a critical role in cellular protection and stress, including response to heat shock. DNAJB2 traffics unfolded proteins to another heat shock protein, HSP70, and activates its ATPase activity to result in a correctly folded protein(s). In this study, we aimed to investigate the effects of the heterozygous DNAJB2 c.823+6C>T mutation on the stress response of DNAJB2 in fibroblasts obtained from the neuropathy patient. **Methods:** The fibroblasts were subjected to one hour of heat shock at 42 °C, and the time course of expression levels of DNAJB2 was established. Additionally, we evaluated the therapeutic efficacy of Cystamine, which has been shown to modulate DNAJB2 levels in cellular and animal models of Huntington’s disease. **Results:** Our results revealed reduced baseline levels of DNAJB2 between the mutant and control fibroblasts. Importantly the mutant cells exhibited a diminished response to heat shock. Thus, the mutation affects the upregulation of DNAJB2 under stress, possibly contributing to the pathogenesis of sensory–motor polyneuropathy. A 48-h pretreatment with 150 μM of Cystamine increased the levels of DNAJB2 in both the control and patient’s fibroblasts. **Conclusions:** To the best of our knowledge, this is the first study to explore this mutant form of DNAJB2 in neuropathy. The study demonstrated that the heterozygous DNAJB2 c.823+6C>T mutation leads to impaired DNAJB2 response to heat shock in the fibroblasts. Cystamine showed promise in restoring DNAJB2 expression, highlighting the need for further research into targeted therapeutic strategies for DNAJB2-related disorders.

## 1. Introduction

Here, we report a 56-year-old male patient diagnosed with adult-onset, severe, sensory–motor polyneuropathy caused by a unique heterozygous *DNAJB2* c.823+6C>T mutation. The *DNAJB2* gene has 10 exons and is expressed in two isoforms. Transcript a contains exons 2–10, while transcript b contains exons 2–9 [1]. The mutation is predicted to affect the two transcripts differently. For transcript a, the mutation is in a splice site, and algorithms predict that it will strengthen the splice site, possibly causing aberrant splicing. For transcript b, the mutation falls in the coding sequence and will lead to an amino acid change from arginine to tryptophan at position 277 of the amino acid sequence. Arginine is highly conserved among species at that site, indicating a potential significance of this mutation (Figure S1).

Mutations in DNAJB2 have been linked to neuromuscular diseases such as distal hereditary motor neuropathy (dHMN) and Charcot-Marie-Tooth disease type 2 (CMT2) [1,2,3,4,5]. These mutations often result in a loss of DNAJB2 expression, leading to peripheral axonal neuropathy with symptoms typically appearing in the second decade of life [1,2,3,4,5]. Some patients also exhibit central nervous system symptoms, including early-onset Parkinsonism [4,5,6]. Moreover, in an in vitro model, it has been shown that these mutations disrupt DNAJB2 expression in fibroblasts, with a presumed similar effect in neurons [1,2]. The exact client proteins and cellular processes affected remain to be determined. Overall, DNAJB2 plays a crucial role in protein quality control, particularly in neurons, by directing misfolded or aggregated proteins to degradation pathways, thereby maintaining cellular homeostasis.

**Figure 1:**
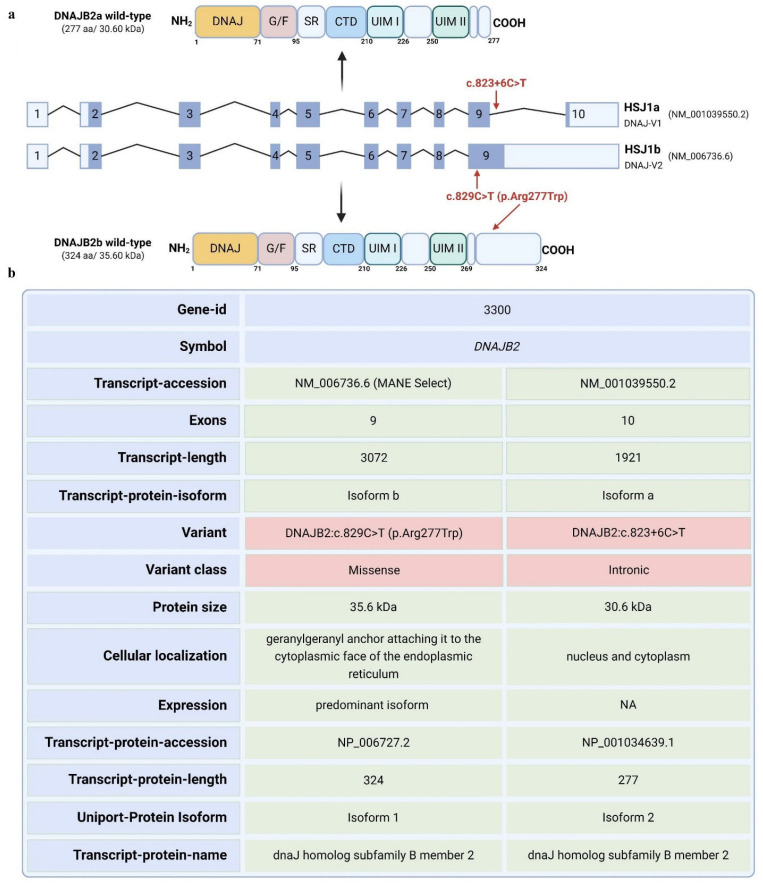
Schematic representation of the human *DNAJB2* gene exons, resultant DNAJB2 protein isoforms and the mutation affecting the protein region. (**a**). The figure depicts the *DNAJB2* gene, highlighting two transcript variants (NM_006736.6 and NM_001039550.2) corresponding to protein isoforms b and a, respectively. The figure also highlights the potential regions in the transcript and the resulting protein that may be impacted by the mutation. (**b**). The table presents key attributes of the transcripts, including the number of exons, transcript lengths, and the resulting protein isoforms. A missense mutation (c.829C>T, p. Arg277Trp) is identified in Isoform b. In contrast, Isoform a contains an intronic variant (c.823+6C>T). The respective protein products exhibit molecular weights of 35.6 kDa for Isoform b and 30.6 kDa for Isoform a, with distinct cellular localizations. Isoform b is anchored to the cytoplasmic face of the endoplasmic reticulum, while Isoform a localizes in both the nucleus and cytoplasm. The predominant expression of Isoform b is indicated, which may be relevant to the pathogenic mechanism in this case.

DNAJB2 (HSP40), initially described as HSJ1, belongs to class II (DNAJB subfamily) of J proteins [7]. It contains an N-terminal J-domain followed by a G/F region. J domain is crucial for binding Hsp70 and activating its ATPase activity [8]. A distinctive feature of HSJ1 is its C-terminal region, which includes two ubiquitin interaction motifs (UIMs) that facilitate binding to polyubiquitylated proteins and the proteasome [9,10]. The glycine/phenylalanine-rich region and ubiquitin-interacting motifs (UIMs) also contribute to specific substrate recognition and targeting for degradation whenever necessary [8,10]. HSJ1 undergoes alternative splicing, resulting in two isoforms with different C-termini and subcellular localizations: DNAJB2a (HSJ1a, 277 aa, 31 kDa), which localizes to the cytosol and nucleus, and DNAJB2b (HSJ1b, 324 aa, 36 kDa), which is associated with the cytoplasmic face of the ER via a C-terminal geranylgeranyl anchor (Figure 1) [8].

**Figure 2:**
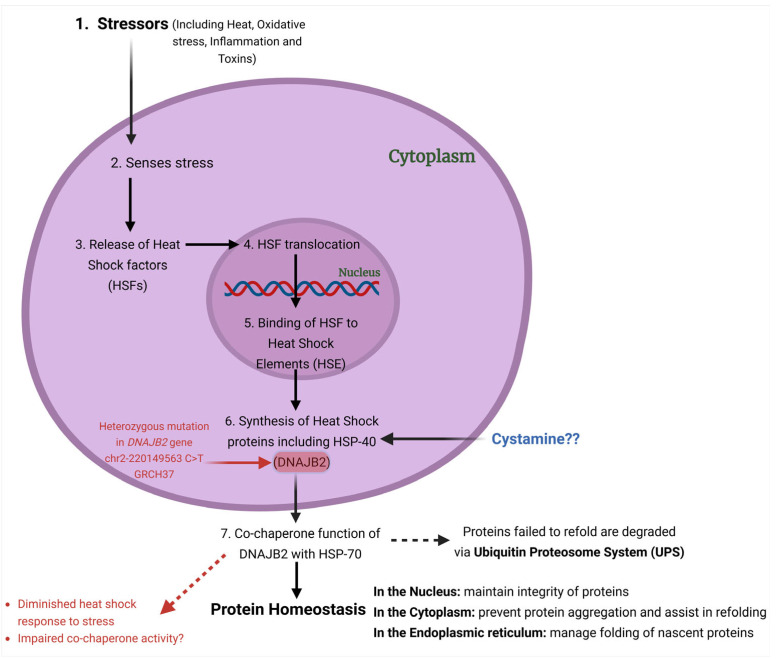
Overview of the heat shock response pathway and the potential effects of the c.823+6C>T mutation on the capacity of DNAJB2 to manage cellular stress mechanisms. 1. Stressors such as heat, oxidative stress, etc., can trigger the heat shock response pathway. 2. The cell senses stress and initiates the heat shock response. 3. Release of Heat Shock Factors (HSFs) from their inactive state in the cytoplasm 4. Activated HSFs translocate to the nucleus. 5. HSFs bind to Heat Shock Elements (HSE), initiating the transcription of heat shock proteins (HSPs). 6. HSPs, including DNAJB2 (HSP40), are synthesized to refold or degrade damaged proteins. 7. DNAJB2 works with HSP70 to maintain protein homeostasis by refolding proteins or directing damaged ones for degradation.

DNAJB2 is predominantly expressed in neurons, particularly in the neocortex, with lower levels detected in other tissues and cells [1,2,7,8]. The primary isoform in neuronal tissues is HSJ1b [1,2,8,11]. DNAJB2 has been reported to be expressed at low levels in cardiac and skeletal muscles as well as fibroblasts [12,13]. Its presence at neuromuscular junctions in mature muscle fibers and its upregulation after eccentric exercise suggest physiological relevance in muscle [12,13]. Functionally, DNAJB2 acts as a co-chaperone for HSPA (HSP70), enhancing HSPA’s ATPase activity and modulating its client binding [14] (Figure 2). Unlike typical co-chaperones that promote protein refolding, DNAJB2 primarily directs proteins towards degradation by the ubiquitin–proteasome system (UPS) [10,15].

In the present study, we aimed to study the effect of the newly identified mutation in our patient on the stress response. We used acute heat shock (at 42 °C) as a source of stress to fibroblasts and assessed the levels of DNAJB2. Additionally, we evaluated the effect of Cystamine on DNAJB2 expression under both baseline and stress conditions in control and mutant DNAJB2 fibroblasts.

Cystamine, an oxidized form of Cysteamine has been reported to increase the levels of isoform HSJ1b in Huntington disease (HD) models [11,16]. Cystamine was found to benefit HD mice by increasing the levels of BDNF in both the brain and serum [11]. The effect was attributed to the inhibition of transglutaminase activity, a process associated with neurodegeneration in HD [17]. However, later studies revealed that the protective effect of Cystamine is independent of transglutaminase inhibition [17]. Other hypotheses include its role in inhibiting caspases, which are involved in late-stage HD pathogenesis [18]. Lastly, Cystamine may modulate cysteine metabolism, increasing antioxidant glutathione (GSH) and hypotaurine levels, further contributing to neuroprotection [18,19]. These various mechanisms highlight the multifaceted role of Cystamine in promoting neuronal survival and function in HD models.

## 2. Results

### 2.1. Patient’s Diagnostic Details

The patient is a 55-year-old man with a history of tingling and burning sensations in the feet for several years, which gradually worsened and were accompanied by significant pain. His exam showed sensory–motor polyneuropathy with length-dependent deficits in vibration, pin prick and touch as well as moderate toe and foot weakness, together with absence of ankle reflexes. He developed a Charcot joint worse on the right foot and needed antibiotic therapy for suspected osteomyelitis. His EMG showed severe axonal, length dependent, sensory motor polyneuropathy.

Genetic screening via a comprehensive neuropathy panel revealed the presence of a heterozygous variant in the *DNAJB2* gene, specifically the c.823+6C>T change, located in intron 9 in transcript a, while in transcript b, it falls in the coding sequence, resulting in an amino acid change at position 277 from arginine to tryptophan (Figure 1a,b). This variant is present in population databases at a very low frequency (rs755166275, ExAC 0.002%). This variant has not been reported in the literature in individuals with DNAJB2-related conditions so far. Nucleotide substitutions within the consensus splice site are a relatively common cause of aberrant splicing [20,21]. Algorithms developed to predict the effect of sequence changes on RNA splicing suggest that this variant may create or strengthen a splice site. The available evidence was insufficient at the time of the genetic report to determine the role of this variant in disease and therefore, it was classified as a Variant of Uncertain Significance.

### 2.2. Sanger Sequencing

Targeted Sanger sequencing of the isoform a transcript revealed no evidence of splicing defects in the region of mutation including intron retention, or any other aberrant splicing events. However, for transcript b, an amino acid change from arginine to tryptophan at position 277 of the amino acid sequence was confirmed.

### 2.3. HSJ1a Expression Is Similar Between Control and Mutant DNAJB2 at Baseline and 24 h Post-Heat Shock Treatment

**Figure 3:**
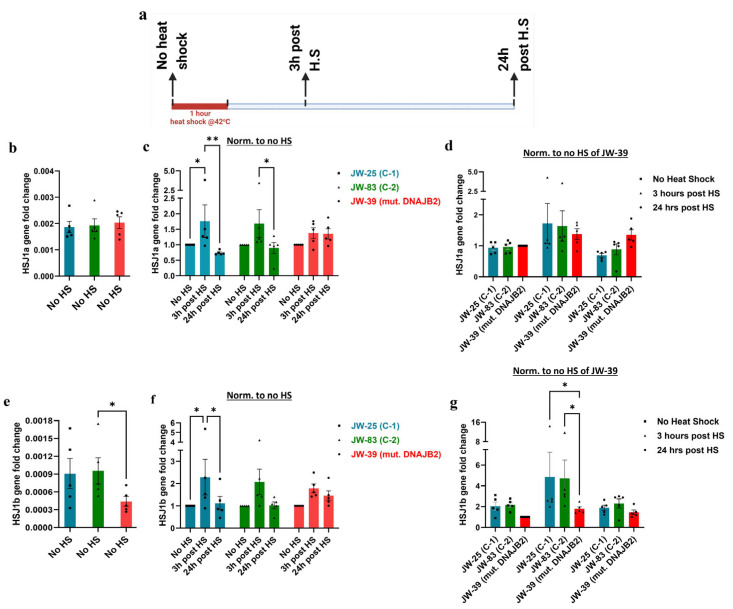
Quantitative PCR analysis of *HSJ1a* and *HSJ1b* gene expression at various time points following a one-hour heat shock. (**a**). Schematic representation of the experimental setup showing the timing of heat shock and subsequent sampling for gene expression analysis. Gene expression levels in all the groups were normalized to their respective expression of housekeeping gene GAPDH. (**b**). Bar graph depicting the basal expression of the *HSJ1a* gene in control fibroblasts (JWAC-25 and JWAC-83) relative to its expression in DNAJB2-mutated patient fibroblasts (JWAC-39). (**c**). Bar graph illustrating the expression of the *HSJ1a* gene in control (JWAC-25 and JWAC-83) and mut. DNAJB2 fibroblasts at 3 h and 24 h post one-hour heat shock, normalized to their respective baseline expression in non-heat-shocked fibroblasts. (**d**). Bar graph displaying the expression of the *HSJ1a* gene expression in control (JWAC-25 and JWAC-83) and mut. DNAJB2 fibroblasts at 3 h and 24 h post one-hour heat shock, with baseline and treatment conditions for all lines normalized to the baseline expression of the mutant fibroblasts. For (**e**–**g**), cells were treated as in b–d but were now evaluated for *HSJ1b* gene expression. Values are expressed as mean ± SEM. Statistical significance was assessed using the Friedman test with uncorrected Dunn’s test to compare the baseline levels, whereas repeated measures two-way ANOVA with uncorrected Fisher’s LSD was applied for comparing the gene expression of *HSJ1a and HSJ1b* at various time points following a one-hour heat shock (n = 5). Results were considered statistically significant at *p* * < 0.05, ** < 0.01, C-1 = Control 1, C-2 = Control 2, mut. = mutant. Note: (**b**,**e**) are evaluating the basal levels without normalization; (**c**,**f**) are looking at the response of the individual line over time, and (**d**,**g**) are comparing the response at that given time.

We first assessed the impact of the mutation in the HSJ1a isoform at the transcript level under baseline and heat shock conditions. Quantitative PCR analysis revealed no significant differences in the baseline expression of *HSJ1a* gene between mut. DNAJB2 (JWAC-39) fibroblasts and the controls (JWAC-25 and JWAC-83) (Figure 3b). Following heat shock treatment, the HSJ1a transcript levels increased at 3 and stabilized by 24 h post-treatment. However, although not significant, the increase in the transcript level at 3 h was slightly lower in the mutant line compared to the two controls and the levels did not stabilize after 24 h (Figure 3c,d).

Throughout this manuscript, we have used two different methods for normalization. In Figure 3c, we normalize each line with no heat shock condition to follow response of each line with treatment over time. In Figure 3d, we normalized the no heat shock of the mutant line to compare between the mutant and controls. This normalization is continued throughout the manuscript.

### 2.4. HSJ1b Expression Is Lower in Mutant Cells

Next, we examined the baseline and stress expression levels of HSJ1b transcript. The results revealed that the *HSJ1b* gene expression was significantly reduced at baseline in mut. DNAJB2 (JWAC-39) fibroblasts compared to the controls (JWAC-83 and JWAC-25) although the difference was statistically significant only when compared to one of the control lines (JWAC-83) (* *p* < 0.05) (Figure 3e).

After heat shock, *HSJ1b* gene expression showed an overall upward trend across all fibroblast lines 3 h post-heat shock, followed by a decline at 24 h and reached comparable levels to the no heat shock condition. When comparing within lines, the mut. DNAJB2 fibroblasts demonstrated a slightly smaller increase in expression with a fold change of 1.78 at 3 h post-heat shock compared to the control fibroblasts that showed a fold change of 2.28 and 2.07, respectively (Figure 3f). This finding was consistent when compared between the lines, where the mutant fibroblasts showed a significantly lower increase (Figure 3g).

Overall, the results showed that JWAC-39 fibroblasts display a trend of impaired HSJ1b upregulation compared to the control lines.

### 2.5. SiRNA Knockdown of DNAJB2 in Fibroblasts

We also performed siRNA knockdown of DNAJB2 to validate that the bands we are quantifying in the Western blot experiments are indeed corresponding to DNAJB2. Following transfection, the knockdown efficiency was assessed post 48 h at both the protein and RNA levels. qPCR analysis confirmed that the RNA levels of both isoforms of DNAJB2 were affected by the siRNA treatment (Figure S2a,b). Additionally, Western blot analysis revealed faint bands corresponding to the two isoforms of DNAJB2, confirming partial knockdown (Figure S2c,d). Only partial downregulation was achieved due to long half-life of the protein and ongoing fibroblasts cell division. Nevertheless, these results validated the specific bands representing the two DNAJB2 isoforms in the subsequent protein expression experiments with/without treatment.

### 2.6. HSJ1b Protein Expression Is Lower in Mutant Cells and Fails to Show Proportional Response to Heat Shock

Next, we wanted to study the effect of this mutation at the level of protein. We first demonstrated a temporal response of DNAJB2 in the human primary fibroblasts when subjected to heat shock conditions (Figure S3). In the densitometric analysis of the two HSJ1 protein isoforms, we have separately quantified isoform a and the doublet of isoform b. Due to limitations in doublet resolution, it was not possible to quantify the individual components of the HSJ1b isoform.

**Figure 4:**
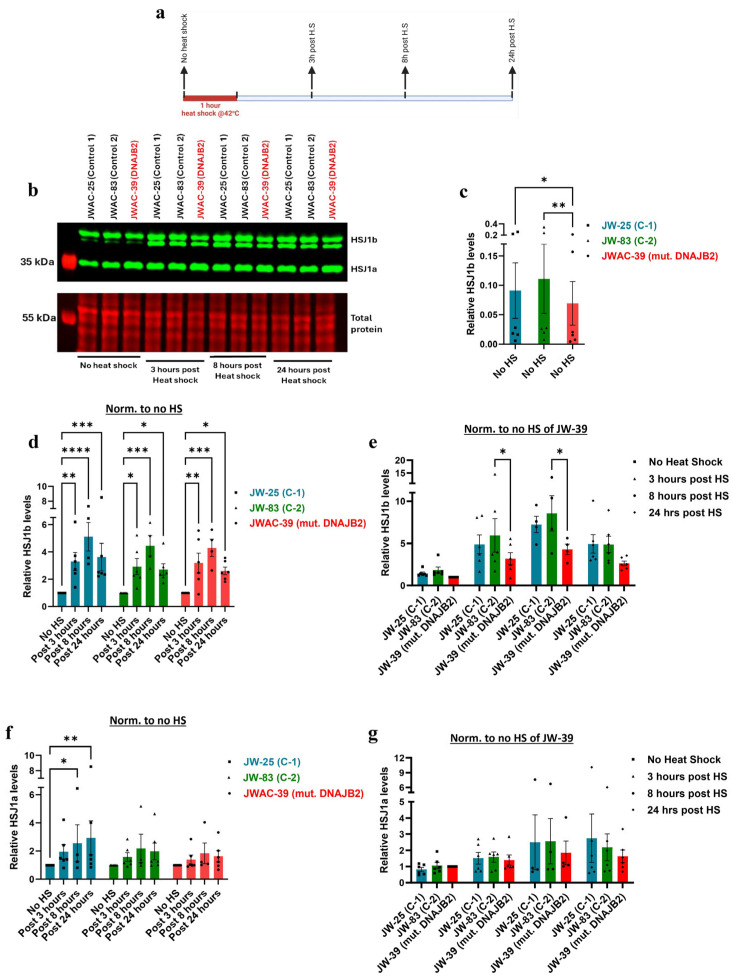
Western blot analysis of DNAJB2 protein levels at various time points following a one-hour heat shock. (**a**). Experimental layout illustrating the timing of heat shock and subsequent sampling for protein expression analysis of HSJ1b. The expression of all the proteins were normalized to their respective total protein. (**b**). Representative immunoblot image displaying the expression of HSJ1a (36 kDa) and HSJ1b (40 kDa) bands in control (JWAC-25 and JWAC-83) and mut. DNAJB2 (JWAC-39) fibroblasts at 0, 3, 8, and 24 h post one-hour heat shock. The lower panel shows their respective total protein stains confirming equal loading of proteins (20 µg/lane). (**c**). Densitometric analysis of the basal expression of HSJ1b protein in the control fibroblasts (JWAC-25 and JWAC−83) relative to those expressed in mut. DNAJB2 patient fibroblasts (JWAC-39). (**d**). Densitometric analysis of HSJ1b protein expression in control (JWAC−25 and JWAC-83) and mut. DNAJB2 fibroblasts at 0, 3, 8, and 24 h post one−hour heat shock, normalized to their respective baseline expression in non-heat-shocked fibroblasts. (**e**). Densitometric analysis of HSJ1b protein expression in control (JWAC-25 and JWAC-83) and mut. DNAJB2 fibroblasts at 0, 3, 8, and 24 h post one-hour heat shock, normalized to the basal expression in mut. DNAJB2 patient fibroblasts. (**f**). Densitometric analysis of HSJ1a protein expression in control (JWAC-25 and JWAC-83) and mut. DNAJB2 fibroblasts at 0, 3, 8, and 24 h post one-hour heat shock, normalized to their respective baseline expression in non-heat-shocked fibroblasts. (**g**). Densitometric analysis of HSJ1a protein expression in control (JWAC-25 and JWAC-83) and mut. DNAJB2 fibroblasts at 0, 3, 8, and 24 h post one-hour heat shock, normalized to the basal expression in mut. DNAJB2 patient fibroblasts. Values are expressed as mean ± SEM. For statistics, Friedman test with uncorrected Dunn’s test was used to compare the baseline levels whereas a mixed-effects model with uncorrected Fisher’s LSD was used to compare the expression of DNAJB2 at various time points following a one-hour heat shock. Results were considered statistically significant at *p* * < 0.05, ** < 0.01, *** < 0.001, **** < 0.0001. (The sample size for DNAJB2 expression is n = 6, except at the 8 h time point (n = 3). C-1 = Control 1, C-2 = Control 2, mut. = mutant. Note: (**c**) is evaluating the basal levels without normalization; (**d**,**f**) is looking at the response of the individual line over time; and (**e**,**g**) are comparing the response at that given time.

Western blot analysis revealed that HSJ1b expression levels were significantly lower in mut. DNAJB2 (JWAC-39) fibroblasts compared to the controls (JWAC-25 and JWAC-83) at baseline (Figure 4b,c). After heat shock treatment, HSJ1b protein levels were significantly increased at 3 and 8 h post-treatment in all fibroblast lines. JWAC-25 and JWAC-83 fibroblasts exhibited fold changes of 3.29 and 2.93, respectively, while the JWAC-39 mutant fibroblasts showed a fold change of 3.18 after 3 h of heat shock (Figure 4d). Similarly, post 8 h of heat shock, JWAC-25 and JWAC-83 fibroblasts displayed fold changes of 5.10 and 4.45, respectively, while JWAC-39 fibroblasts showed a fold change of 4.29 when compared to the no heat shock condition (Figure 4d). However, when comparing between the lines, mutant fibroblasts exhibited a significantly smaller increase compared to control fibroblasts at 3 and 8 h post-heat shock (although the difference was significant with one of the controls, JWAC-83) (Figure 4e).

Additionally, the HSJ1a isoform showed an overall upward trend across all fibroblast lines at 3, 8, and 24 h post-heat shock. When comparing within lines, the mut. DNAJB2 fibroblasts demonstrated a slightly smaller increase in expression with a fold change of 1.40 at 3 h post-heat shock compared to the control fibroblasts that showed a fold change of 1.95 and 1.40, respectively (Figure 4f). However, the difference was statistically significant only when compared to one of the control lines (JWAC-83).

These results suggest that both the control and mutant fibroblasts follow a similar pattern of HSJ1b upregulation after heat shock. However, the lower baseline expression in the mutant cells resulted in a comparatively smaller increase in the HSJ1b levels under stress conditions.

### 2.7. Heat Shock Did Not Affect the Viability of Mut. DNAJB2 and Control Fibroblasts

**Figure 5:**
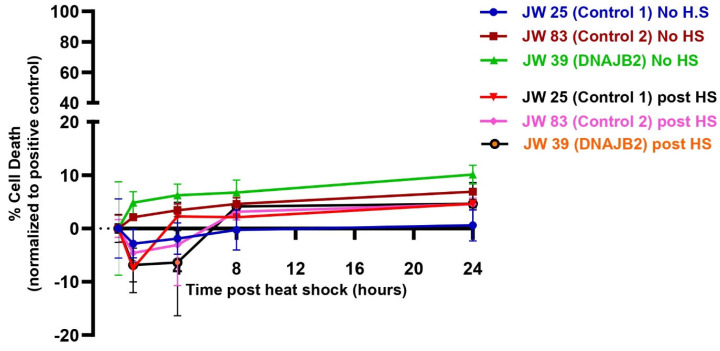
Cell viability assay of control and mut. DNAJB2 fibroblasts post one-hour of heat shock. Fibroblasts were grown in two plates and one of them was subjected to one-hour of heat shock (heat shock plate). CellTox Green Dye was added one day before the heat shock treatment. Fluorescence was measured at 0, 1, 4, 8, 12, 16, 20, and 24 h post heat shock treatment. Percentage of cell death in each line were calculated relative to those caused by 1% Triton X-100 (positive control) set to 100%. Cell death in the control fibroblasts JWAC-25 (Purple), JWAC-83 (Brown) and JWAC-39 (green) in the no heat shock plate and JWAC-25 (black), JWAC-83 (Pink) and JWAC-39 (orange) in the heat shock plate were plotted at different time points post heat shock. Values are expressed as mean ± SD (n = 4, technical replicates). Note: There was an initial dip in the fluorescence intensity immediately following heat shock in the treated groups. This transient dip is attributed to the instability of the CellTox™ Green dye at elevated temperatures, such as 42 °C. The dye’s fluorescence stabilized after approximately 3 h. Therefore, the negative fluorescence values recorded during this period are technical artifacts and should not be interpreted as indicators of cell viability.

We then assessed cell viability in both DNAJB2-mutant and control fibroblasts following heat shock to determine if the mutation increases susceptibility of cells to stress-induced cell death. The results, plotted in Figure 5, showed that cell death in the control fibroblasts (JWAC-25 and JWAC-83) and mut. DNAJB2 fibroblasts (JWAC-39) remained low and comparable across all time points, irrespective of the heat shock conditions. The percentage of cell death was consistent across all fibroblast lines, with no significant increase over time. Therefore, the results showed that the heat shock treatment did not cause cell death across both mut. DNAJB2 and control fibroblasts, suggesting that, mut. DNAJB2 fibroblasts do not have an increased susceptibility to cell death under heat shock conditions compared to controls (n = 4, technical replicates).

### 2.8. Cystamine Elicits an Effect on Basal DNAJB2 Expression in Control and Mut. DNAJB2 Fibroblasts

**Figure 6:**
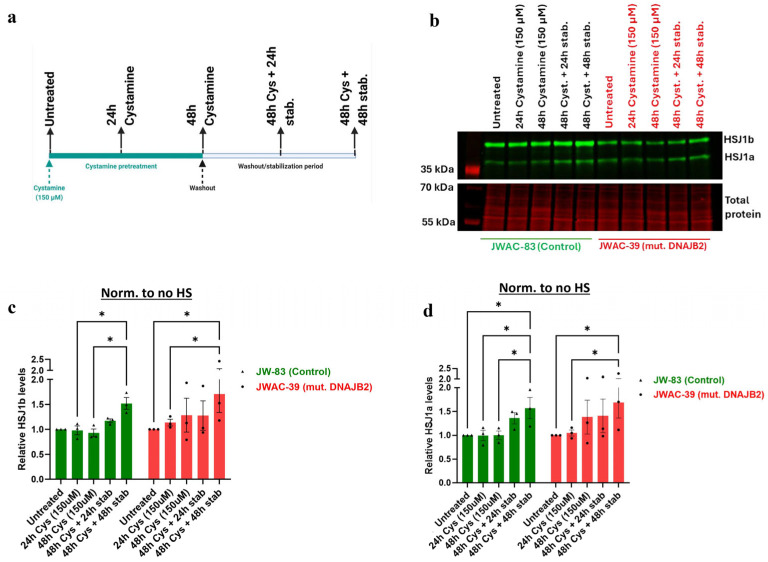
Effect of Cystamine pretreatment on the levels of DNAJB2 (HSP40). (**a**). Experimental layout illustrating the time of introduction of Cystamine, and a washout period maintained to study its effect on the expression of HSJ1b protein levels. The expression of HSJ1b in different groups were normalized to their respective total protein. (**b**). Representative immunoblot image showing the expression of HSJ1a (36 kDa) and HSJ1b (40 kDa) bands in the controls (JWAC-83) and mut. DNAJB2 (JWAC-39) fibroblasts, detected with anti-DNAJB2 antibody across different exposure times and washout periods of Cystamine (150 µM). The lower panel represents their respective total protein stains, confirming comparable loading of proteins (20 µg/lane). (**c**). Densitometric analysis of the expression of HSJ1b isoform protein in control (JWAC-83) and mut. DNAJB2 (JWAC-39) fibroblasts across different exposure times and washout periods of Cystamine, normalized to the basal expression of the protein in their respective untreated fibroblasts. (**d**). Densitometric analysis of the expression of HSJ1a isoform proteins in control (JWAC-83) and mut. DNAJB2 (JWAC-39) fibroblasts across different exposure times and washout periods of Cystamine, normalized to the basal expression of the protein in their respective untreated fibroblasts. Values are expressed as mean ± SEM. For statistics, repeated measures two-way ANOVA with uncorrected Fisher’s LSD were used. Results were considered statistically significant at *p* * < 0.05 (n = 3 for DNAJB2 expression). (Stab. = Stabilization).

Next, we explored the ability of Cystamine to induce or restore normal DNAJB2 expression in both control and mutant fibroblasts. The results demonstrated that pretreatment with 150 μM of Cystamine significantly increased both HSJ1a and HSJ1b expression in control and mutant fibroblasts (Figure 6b–d). Specifically, after 48 h of Cystamine pre-treatment followed by a 48-h washout, the relative expression of HSJ1b was significantly higher than in the untreated cells.

Overall, Cystamine pretreatment effectively enhanced DNAJB2 expression in both control and mutant DNAJB2 fibroblasts, and the mutation has not impaired the ability of the cells to increase DNAJB2 levels with Cystamine pretreatment.

## 3. Discussion

In this work, we explored the effect of a heterozygous *DNAJB2* c.823+6C>T mutation on the RNA and protein expression levels. We evaluated the ability of mutant cells to appropriately upregulate expression levels of both the isoforms of DNAJB2 after heat shock and upon Cystamine stimulation. Our results are impacted by working with a heterozygous and not a homozygous cell line, and it is possible that the effects of the mutation in a homozygous cell line would have been more pronounced and easier to delineate. Given that the patient has developed disease despite the presence of one normal allele, it was justifiable to use un-manipulated fibroblasts instead of an engineered homozygous cell line. Although, this is not the best cell line, not a neuronal cell, the ease of obtaining, maintaining, and stimulating fibroblasts with heat shock determined our choice.

Mutations in the *DNAJB2* gene have been linked to various neurodegenerative disorders [22]. Different specific mutations are associated with distinct neurological conditions [22]. For example, mutations that affect splicing at introns 4 and 5 of the gene are reported to cause distal hereditary motor neuropathy, indicating that accurate splicing is crucial for normal protein function. Another mutation in the J-domain of DNAJB2 is associated with Charcot Marie Tooth disease type 2 (CMT-2), which causes progressive muscle weakness. Additionally, splice site deletion and a deletion affecting the J-domain have been found in patients with severe motor neuron diseases like spinal muscular atrophy and juvenile Parkinsonism [1,2,3,5,23]. These findings highlight the vital role of DNAJB2 in maintaining neuronal integrity and its implication in these debilitating conditions.

The c.823+6C>T variant identified in the *DNAJB2* gene in this neuropathy patient is located within the splice site of intron 9. This type of mutation typically results in abnormal splicing, leading to either the production of aberrant proteins or a reduction in functional protein levels. While in silico predictions suggest that this mutation could potentially affect RNA splicing, its direct role in the pathogenesis of DNAJB2-related conditions is still uncertain, as no prior studies have documented the pathogenicity of this specific variant. Since this variant has contributed to the polyneuropathy observed in the patient and this protein generally has a crucial role in protein quality control and cellular stress response, we examined the exact impact of this variant on the expression levels of DNAJB2 under normal and stressful conditions.

Our findings revealed that at baseline, *HSJ1a* gene expression was not significantly different between the DNAJB2-mutated fibroblasts and the controls (Figure 3b). This suggests that the mutation did not affect HSJ1a expression under non-stressed conditions. The HSJ1a transcripts did show an increase in expression at 3 h post-heat shock, with no observable differences between control and mutant cells (Figure 3d). This suggests that the mutation does not appear to have a substantial effect on HSJ1a expression or its regulation in response to heat stress. However, *HSJ1b* gene expression was significantly lower at baseline in mut. DNAJB2 fibroblasts compared to controls, suggesting that the mutation specifically affects the expression of this isoform (Figure 3e). After heat shock treatment, HSJ1b expression remained low in the mut. DNAJB2 fibroblasts compared to the control fibroblasts (Figure 3g). In our patient, although we do not have direct evidence of the effect of the mutation on neuronal cells, the evidence from fibroblasts suggests that a possible reduction in the baseline level of HSJ1b as well as under stress conditions might lead to misfolded protein accumulation due to insufficient folding or failure to direct these proteins to the proteasome.

Supporting these findings, immunoblot analysis revealed that HSJ1b protein expression was significantly lower at baseline in the mutated fibroblasts, and the increase in response to heat shock was substantially weaker compared to the controls (Figure 4b,c,e). Post-translational geranylgeranylation is essential for the C-terminal end of HSJ1b to mediate its attachment to the cytoplasmic side of the endoplasmic reticulum membrane. Therefore, the mutation that falls in the C-terminal end of the protein, such as in our case, may have prevented proper attachment, thereby disrupting its normal localization and expression [24]. While the magnitude of the response in DNAJB2-mutated cells was similar to that of the two control lines following heat shock, the lower baseline level of HSJ1b expression in these cells did not reach the abundance levels, as seen in the control cells (Figure 4b,d,e).

Although outside of the scope of this work, implications from our findings may relate to neuronal processes which will be investigated in future studies. DNAJB2 plays a critical role in protein quality control by directing misfolded or aggregated proteins to the ubiquitin–proteasome system for degradation, thereby preventing toxic accumulation within neurons [25]. he expression of DNAJB2 in peripheral nerve axons alongside neurofilaments indicates that the mutation in this protein could disrupt axonal transport [12]. Similarly, the abundant expression of DNAJB2 at the neuromuscular junction (NMJ), where it interacts with other chaperones involved in facilitating the clustering of acetylcholine receptors, suggests possible defects in NMJ maintenance [26]. Overall, our findings highlight the significant implication of HSJ1b dysfunction both at RNA and protein level. Mutation resulting in impaired DNAJB2 function might have led to the accumulation of misfolded proteins, disrupted axonal transport, and compromised NMJ integrity, collectively contributing to the pathological process.

Despite the impaired cellular response to heat shock, the level of cell death was even between DNAJB2-mutated and control fibroblasts across all conditions (Figure 5). In the heat shock group, we observed an initial dip in fluorescence intensity immediately following heat shock, which stabilized after approximately 3 h. This fluctuation is technical, likely caused by the instability of the CellTox dye under heat shock conditions, rather than indicating actual changes in cell viability. While the mutation affected the expression of DNAJB2, it did not increase susceptibility to cell death under stress conditions. This indicates that the cellular dysfunction caused by the mutation is sufficiently balanced by the product of the normal allele. Next, we found that Cystamine (150 µM) pretreatment significantly increased DNAJB2 expression in both control and mut. DNAJB2 fibroblasts (Figure 6b,c). This suggests that Cystamine may restore DNAJB2 expression, potentially offering a therapeutic strategy for enhancing the levels of this protein. Future studies investigating its precise role in BDNF up-regulation, caspase inhibition, and cysteine metabolism would provide deeper insights into its therapeutic potential in DNAJB2-related disorders.

## 4. Materials and Methods

### 4.1. Recruitment of Patients

The patient (JWAC-39) was clinically evaluated by a Neuromuscular Neurologist (Dr. Hristelina Ilieva) at Saint Luke’s Hospital, Kansas City (Table 1) and diagnosed with severe sensory motor polyneuropathy. Genetic neuropathy panel revealed a *DNAJB2* c.823+6C>T mutation. The skin punch of the patient was obtained by the Weinberg ALS Center, Thomas Jefferson University and fibroblast lines were established. Fibroblast cultures were also obtained from two age-matched healthy controls (JWAC-25 and JWAC-83) for comparison.

### 4.2. Sanger Sequencing

Total RNA was extracted from patient fibroblasts using TRIzol (Invitrogen, Carlsbad, California, Catalog: 15596018) and stored at −80 °C till further analysis. The extracted samples were transported on dry ice for commercial Sanger sequencing to Azenta, South Plainfield, NJ, USA (formerly GENEWIZ), which included PCR clean-up and bidirectional sequencing of both the isoforms of DNAJB2. The primers were designed to cover the mutation site of both the isoforms from both the directions. All primers were synthesized by Genewiz and are listed in Supplementary Table S2. Chromatograms were analyzed using Chromas version 2.6.6 to identify potential splicing events, such as exon or the intron retention of the transcript, caused by the variant.

### 4.3. Collection and Culturing of Fibroblasts

After obtaining informed consent from the patient/healthy volunteers, a 3 mm skin punch biopsy was performed on each participant after local anesthesia. The collected skin biopsies were seeded in a 10 cm dish containing Dulbecco’s Modified Eagle Medium (DMEM), high glucose (Cytiva, Marlborough, MA, USA, Cat. SH30243.01) supplemented with 20% fetal bovine serum (FBS) (Gibco, Jenks, OK, USA, Catalog: 10437028), and 1% penicillin-streptomycin (HyClone, Catalog: SV30010). The dishes were incubated at 37 °C in a humidified atmosphere containing 5% CO_2_. Fibroblasts were allowed to grow, and media was changed every 2–3 days until the growing fibroblast cultures reached confluence. The cells were frozen in liquid nitrogen, and the subsequent passages from 3 to 6 were used for the experiments.

### 4.4. Heat Shock Treatment

To induce a heat shock response, fibroblast cultures were exposed to 42 °C for 1 h in a CO_2_ incubator, followed by a recovery period at 37 °C at various time points (3, 8, and 24 h post heat shock). The expressions were compared to the cells without undergoing heat shock treatment. This temperature (42 °C) has been reported to be effective in inducing heat shock response, including the upregulation of heat shock proteins such as HSP40 and HSP70, without causing immediate cell death [27,28,29]. The levels of DNAJB2 were assessed via Western blot at these time points to determine the trajectory of the heat shock response. The optimal time points for subsequent treatments were selected based on the maximal expression levels of the protein.

### 4.5. Cystamine Treatment

For Cystamine treatment, fibroblasts were incubated with 150 µM Cystamine for 24 and 48 h. This dose was based on a previous study that used 100 µM in mouse immortalized neuronal cells demonstrating its effectiveness in increasing HSJ1b levels [11]. In our preliminary experiments, a 150 µM dose was found to be more effective in eliciting the desired response. To allow sufficient time for DNAJB2 protein production, cells were harvested after a 48 h incubation period following treatment (Figure S4a,b).

### 4.6. CellTox Assay

To determine cell viability post heat shock, the CellTox™ Green Cytotoxicity Assay was employed. Initially, the CellTox™ Green Dye was completely thawed in a 37 °C water bath, mixed using a vortex mixer for homogeneity, and briefly centrifuged to collect the dye at the bottom of the tube. Cells were harvested and adjusted to the desired density, specifically 7500 cells per 100 µL of fresh cell culture medium. Subsequently, 10 µL of the CellTox™ Green Dye was added to each 5 mL of cell suspension, mixed by inversion or gentle vortexing to ensure even distribution of the dye. The cell–dye mixture was then pipetted into a sterile multiwell plate at 100 µL per well for a 96-well format and allowed to adhere overnight.

The following day, cells were subjected to a 1 h heat shock at 42 °C. For positive control wells, Lysis Solution was added at a ratio of 1:25 (4 µL per 100 µL of cells) to represent the maximum signal obtainable from the lysed cells. Fluorescence intensity was measured at intervals between 0 and 24 h post-heat shock using an excitation wavelength of 485–500 nm and an emission wavelength of 520–530 nm. Prior to each reading, the plate was shaken for one minute at 700–900 rpm to ensure homogeneity and was returned to the incubator between readings. The fluorescence readings (RFU) were plotted against time to assess the cytotoxic effects of the heat shock treatment on the cells.

### 4.7. RNA Isolation and PCR Analysis

Cells were collected directly in TRIzol (Invitrogen, Carlsbad, CA, USA, Catalog: 15596018) and total RNA was isolated following the manufacturer’s protocol. Complementary DNA (cDNA) was synthesized from the isolated RNA using the QuantiTect Reverse Transcription Kit (Qiagen, Germantown, MD, USA, Catalog: 205311). Quantitative PCR (qPCR) was performed using the PowerUp™ SYBR™ Green Master Mix (Thermo Fisher, Waltham, MA, USA, Catalog: A25742) according to the manufacturer’s instructions.

We used splice junction primers that could clearly distinguish isoform a from b. HS1a composed of exons 2 to 10 whereas HS1b composed of exons 2 to 9 (missing exon 10). Therefore, HS1a primers amplify the junction between exon 9 and exon 10 whereas HS1b primers amplify the extended region of exon 9 that is not present in HSJ1a.

For each qPCR reaction, 30 ng of cDNA was used. The experiments were performed in duplicates across 5 independent RT-qPCR experiments using cell lines from different passages of both patient and control samples. The purified RNA samples were first incubated with gDNA Wipeout Buffer to effectively eliminate DNA contamination. Moreover, we performed no template control (NTC) of the primers to ensure no contamination and primer-dimer formation. The amplification cycles of specific transcripts were normalized to the amplification cycles of the housekeeping gene, GAPDH. The cycle threshold (Ct) values were analyzed using the ThermoFisher Connect Platform to determine the relative fold change in gene expression. The primers used for all the transcripts are listed in Table S1 [5].

### 4.8. Protein Extraction and Western Blot Analysis

Following heat shock and/or treatment with Cystamine, cells from each well of a 6-well plate were scraped using 50 µL of radioimmunoprecipitation assay (RIPA) buffer (50 mM Tris-HCl pH 8.0, 1 mM EDTA, 1% Triton X-100, 0.1% SDS, and 150 mM NaCl). The RIPA buffer was supplemented with 1x Halt™ Protease Inhibitor Cocktail (EDTA-Free, 100X, Cat. 87785, Thermo Fisher Scientific, Waltham, MA, USA) and 1x Halt™ Phosphatase Inhibitor Cocktail (100X, Cat. 78420, Thermo Fisher Scientific, Waltham, MA, USA).

Cells were incubated on ice for 5 min, followed by sonication at 4 °C for 10 cycles (30 s on, 30 s off) using the Diagenode Pico Bioruptor Sonication System with MiniChiller 300. The sonicated extracts were then centrifuged at 21,000× *g* for 10 min at 4 °C. The supernatant was collected and used for subsequent experiments. Protein concentration of the extracts was determined using the Pierce™ BCA Protein Assay Kit (Catalog: A55865, Thermo Fisher Scientific, Waltham, MA, USA).

For Western blot analysis, 20 µg of protein were loaded and separated by 12% SDS-PAGE, followed by transfer to nitrocellulose membrane with the Trans-Blot Turbo system (Bio-Rad, Hercules, CA, USA). Total protein was stained with the Revert 700 Total Protein Stain (Li-Cor Biosciences, Lincoln, NE, USA) and scanned with the Odyssey scanner (Li-Cor Systems, Lincoln, NE, USA). The membranes were then blocked with 5% non-fat dry milk (Fisher Scientific, Waltham, MA, USA, Catalog: NC9871209). The blots were then probed with the following primary antibodies: DNAJB2 Polyclonal antibody (Proteintech, Rosemont, IL, USA, Catalog: 10838-1-AP, RRID: AB_2277491). Secondary antibodies: Goat anti-Rabbit IgG (H+L) Alexa Fluor™ Plus 800 ((Thermo Fisher Scientific, Waltham, MA, USA, Catalog: A32735, RRID: AB_2633284). Both the primary and secondary antibodies were diluted in 3% Bovine serum albumin and 0.2% Tween. Band intensities were measured using Image Studio software version 2.1.15 (Li-Cor Systems, Lincoln, NE, USA). Bands of interest were captured with intensity measured, and comparably sized adjacent regions were captured to reflect mean levels of background intensity for background normalization. Normalized values, with respect to total protein, were used to determine the relative upregulation or downregulation of target proteins. Each experiment was repeated at least three times to ensure reproducibility. Fold changes in protein expression in treated fibroblasts were calculated relative to untreated controls for the Cystamine experiment.

In the densitometric analysis of the two DNAJB2 protein isoforms, we have separately quantified isoform a and the doublet of isoform b. Due to limitations in doublet resolution, it was not possible to quantify the individual components of the HSJ1b isoform.

### 4.9. SiRNA Knockdown of DNAJB2 in Fibroblasts

To ensure the specificity of the bands detected in Western blot analysis, the *DNAJB2* gene was knocked down in fibroblasts using small interfering RNAs (siRNAs). The siRNA transfection process involved using specific siRNA sequences targeting exon 3 and 4 of *DNAJB2* gene (DNAJB2 Silencer™ Select Pre-Designed siRNA, Thermofisher, Waltham, MA, USA, assay ID: s6959) using P2 Primary Cell 4D X Kit L (24 RCT) (Lonza Bioscience, Walkersville, MD, USA, Catalog: V4XP-2024) following the manufacturers protocol, the siRNA was incubated for 48 h to ensure for efficient downregulation.

The validation of the knockdown was assessed through qPCR, measuring the expression levels of the two *DNAJB2* gene isoforms. Similarly, the effects of the knockdown at the protein level were analyzed using Western blotting.

### 4.10. Statistical Analysis

All the values of protein expression in control and mutant DNAJB2 (mut. DNAJB2) fibroblasts were normalized to the basal expression levels of the respective proteins in the mut. DNAJB2 fibroblasts to allow for comparison of the basal differences between the lines and the level of expression under different experimental conditions.

Data were analyzed using GraphPad Prism 10.2.3 version software. To compare within the group, the heat-shock response after heat shock was normalized to its respective baseline expression in both RNA and protein experiments. To compare the fold change in RNA/protein expression to the heat shock response across groups, the baseline and treatment conditions for all lines were normalized to the baseline expression of the mutant fibroblasts. Statistical comparisons between groups to assess baseline protein expression were performed using the Friedman test with uncorrected Dunn’s test. For comparisons evaluating the effect of heat shock, a mixed-effects model with uncorrected Fisher’s LSD was used for experiments with missing values at some time-points, while repeated measures two-way ANOVA with uncorrected Fisher’s LSD was applied for experiments with no missing values. Results were considered statistically significant at *p* * = <0.05, ** = <0.01, *** = <0.001, **** = <0.0001. Data in the graphs are presented as mean ± SEM.

## 5. Conclusions

Our study demonstrates that the c.823+6C>T variant in the *DNAJB2* gene is associated with impaired expression of DNAJB2 in fibroblasts under stress. As this mutation is heterozygous, it has affected only one DNAJB2 allele, resulting in partial disruption of DNAJB2 expression and function. DNAJB2-mutated cells demonstrated lower HSJ1b expression at baseline. While they mounted a robust stress response upon stimulation via heat shock, their response did not reach the levels observed in control cells. While the effects in fibroblasts are modest, a cell like the neuron that lives for many years in humans accumulates a significant amount of changed proteins and needs to continuously fold them appropriately. Thus, even small alterations in protein folding capacity could be detrimental. For example, when exposed to stressors like injury or oxidative stress, both sensory and motor neurons can upregulate HSPs as a protective mechanism, but the magnitude of this response is higher in sensory neurons [30]. Thus, a mutation in the heat shock protein makes sensory neurons potentially more vulnerable to stress-induced damage, potentially contributing to the disease’s pathological processes. While our findings suggest a potential therapeutic role for Cystamine in enhancing DNAJB2 levels, further investigation is necessary to explore its mechanism and optimize its efficacy, particularly under stress conditions.

Future studies should explore the underlying mechanisms of impaired upregulation of DNAJB2. Additionally, the impact of the mutation on specific client proteins should be investigated using a technique that identifies protein interactions, like Proximity Ligation assays combined with sequencing. Identifying novel therapeutic targets to restore protein homeostasis in DNAJB2-mutated cells remains a crucial area of research.

## Figures and Tables

**Table 1:** Patient details whose fibroblasts were used for the experiments.

Patients	Age at Examination (Years)/Sex	Disease Diagnosed	Motor Symptoms
JWAC-39	56/M	Neuropathy patient carrying *DNAJB2* mutation	Yes
JWAC-25	59/M	Healthy volunteer	No
JWAC-83	59/M	Healthy volunteer	No

## Data Availability

The original contributions presented in this study are included in the article. Further inquiries can be directed to the corresponding author.

## Supplementary Materials

The following supporting information can be downloaded at: https://www.mdpi.com/article/10.3390/neurolint17050073/s1. Figure S1: Shows the c.823+6C>T mutation site within the DNAJB2 gene on chromosome 2, highlighting the specific nucleotide change caused by the mutation. Figure S2: siRNA knockdown of DNAJB2 on the expression of DNAJB2 and HSP70. Figure S3: Expression of DNAJB2 (HSP40) at different time-points post one-hour of heat shock. Figure S4: Effect of Cystamine pretreatment on the levels of DNAJB2 (HSP40) at different time-points. Table S1: Primers used for different transcripts in the experiments. Table S2: Primers used for Sanger Sequencing.

## Abbreviations

The following abbreviations are used in this manuscript:

ALS	Amyotrophic Lateral Sclerosis
ATPase	Adenosine Triphosphatase
BDNF	Brain-Derived Neurotrophic Factor
CMT2	Charcot-Marie-Tooth disease type 2
DMEM	Dulbecco’s Modified Eagle Medium
DNAJB2 (HSP40)	A member of the heat shock protein family, often referred to as Heat Shock Protein 40
EMG	Electromyography
ER	Endoplasmic Reticulum
FBS	Fetal Bovine Serum
G/F region	Glycine/Phenylalanine-rich region
GSH	Glutathione
HSP70	Heat Shock Protein 70
HSPs	Heat Shock Proteins
J-domain	A domain in J proteins responsible for interaction with Hsp70 proteins
NMJ	Neuromuscular Junction
PCR	Polymerase Chain Reaction
qPCR	Quantitative Polymerase Chain Reaction
RIPA	Radioimmunoprecipitation Assay
SEM	Standard Error of the Mean
siRNA	Small Interfering RNA
UPS	Ubiquitin–Proteasome System
UIMs	Ubiquitin Interaction Motifs
RNA	Ribonucleic Acid
DNA	Deoxyribonucleic Acid

## References

[1] Blumen S.C., Astord S., Robin V., Vignaud L., Toumi N., Cieslik A., Achiron A., Carasso R.L., Gurevich M., Braverman I. (2012). A rare recessive distal hereditary motor neuropathy with HSJ1 chaperone mutation. Ann. Neurol..

[2] Gess B., Auer-Grumbach M., Schirmacher A., Strom T., Zitzelsberger M., Rudnik-Schöneborn S., Röhr D., Halfter H., Young P., Senderek J. (2014). HSJ1-related hereditary neuropathies: Novel mutations and extended clinical spectrum. Neurology.

[3] Lupo V., García-García F., Sancho P., Tello C., García-Romero M., Villarreal L., Alberti A., Sivera R., Dopazo J., Pascual-Pascual S.I. (2016). Assessment of Targeted Next-Generation Sequencing as a Tool for the Diagnosis of Charcot-Marie-Tooth Disease and Hereditary Motor Neuropathy. J. Mol. Diagn..

[4] Frasquet M., Chumillas M.J., Vílchez J.J., Márquez-Infante C., Palau F., Vázquez-Costa J.F., Lupo V., Espinós C., Sevilla T. (2016). Phenotype and natural history of inherited neuropathies caused by *HSJ1* c.352+1G & A mutation. J. Neurol. Neurosurg. Psychiatry.

[5] Sanchez E., Darvish H., Mesias R., Taghavi S., Firouzabadi S.G., Walker R.H., Tafakhori A., Paisán-Ruiz C. (2016). Identification of a Large *DNAJB2* Deletion in a Family with Spinal Muscular Atrophy and Parkinsonism. Hum. Mutat..

[6] Teive H., Kok F., Raskin S., Arruda W. (2016). Distal hereditary motor neuropathy with HSJ1 chaperone mutation, presenting with peripheral motor neuropathy, associated to parkinsonism, and cerebellar ataxia. Case report. Park. Relat. Disorders.

[7] Cheetham M.E., Brion J.P., Anderton B.H. (1992). Human homologues of the bacterial heat-shock protein DnaJ are preferentially expressed in neurons. Biochem. J..

[8] Chapple J.P., Cheetham M.E. (2003). The Chaperone Environment at the Cytoplasmic Face of the Endoplasmic Reticulum Can Modulate Rhodopsin Processing and Inclusion Formation. J. Biol. Chem..

[9] Kampinga H.H., Craig E.A. (2010). The HSP70 chaperone machinery: J proteins as drivers of functional specificity. Nat. Rev. Mol. Cell Biol..

[10] Westhoff B., Chapple J.P., Van Der Spuy J., Höhfeld J., Cheetham M.E. (2005). HSJ1 Is a Neuronal Shuttling Factor for the Sorting of Chaperone Clients to the Proteasome. Curr. Biol..

[11] Borrell-Pages M. (2006). Cystamine and cysteamine increase brain levels of BDNF in Huntington disease via HSJ1b and transglutaminase. J. Clin. Investig..

[12] Claeys K.G., Sozanska M., Martin J.-J., Lacene E., Vignaud L., Stockholm D., Laforêt P., Eymard B., Kichler A., Scherman D. (2010). DNAJB2 Expression in Normal and Diseased Human and Mouse Skeletal Muscle. Am. J. Pathol..

[13] Mahoney D.J., Safdar A., Parise G., Melov S., Fu M., Macneil L., Kaczor J., Payne E.T., Tarnopolsky M.A. (2008). Gene expression profiling in human skeletal muscle during recovery from eccentric exercise. Am. J. Physiol.-Regul. Integr. Comp. Physiol..

[14] Cheetham M.E., Jackson A.P., Anderton B.H. (1994). Regulation of 70-kDa heat-shock-protein ATPase activity and substrate binding by human DnaJ-like proteins, HSJ1a and HSJ1b. Eur. J. Biochem..

[15] Howarth J., Kelly S., Keasey M., Glover C., Lee Y.B., Mitrophanous K., Chapple J., Gallo J., Cheetham M., Uney J. (2007). Hsp40 Molecules That Target to the Ubiquitin-proteasome System Decrease Inclusion Formation in Models of Polyglutamine Disease. Mol. Ther..

[16] Arbez N., Roby E., Akimov S., Eddings C., Ren M., Wang X., Ross C.A. (2019). Cysteamine Protects Neurons from Mutant Huntingtin Toxicity1. J. Huntington’s Dis..

[17] Bailey C.D., Johnson G.V. (2006). The protective effects of cystamine in the R6/2 Huntington’s disease mouse involve mechanisms other than the inhibition of tissue transglutaminase. Neurobiol. Aging.

[18] Lesort M., Lee M., Tucholski J., Johnson G.V.W. (2003). Cystamine Inhibits Caspase Activity. J. Biol. Chem..

[19] Paul B.D., Snyder S.H. (2019). Therapeutic Applications of Cysteamine and Cystamine in Neurodegenerative and Neuropsychiatric Diseases. Front. Neurol..

[20] Buratti E., Chivers M., Královičová J., Romano M., Baralle M., Krainer A.R., Vořechovský I. (2007). Aberrant 5′ splice sites in human disease genes: Mutation pattern, nucleotide structure and comparison of computational tools that predict their utilization. Nucleic Acids Res..

[21] Zhang M. (1998). Statistical features of human exons and their flanking regions. Hum. Mol. Genet..

[22] Zarouchlioti C., Parfitt D.A., Li W., Gittings L.M., Cheetham M.E. (2018). DNAJ Proteins in neurodegeneration: Essential and protective factors. Philos. Trans. R. Soc. B Biol. Sci..

[23] Gonzaga-Jauregui C., Harel T., Gambin T., Kousi M., Laurie, Francescatto L., Ozes B., Karaca E., Jhangiani S.N., Matthew (2015). Exome Sequence Analysis Suggests that Genetic Burden Contributes to Phenotypic Variability and Complex Neuropathy. Cell Rep..

[24] Sarparanta J., Jonson P.H., Reimann J., Vihola A., Luque H., Penttilä S., Johari M., Savarese M., Hackman P., Kornblum C. (2023). Extension of the DNAJB2a isoform in a dominant neuromyopathy family. Hum. Mol. Genet..

[25] Saveri P., Magri S., Maderna E., Balistreri F., Lombardi R., Ciano C., Moda F., Garavaglia B., Reale C., Pinter G.L. (2022). DNAJB2-related Charcot-Marie-Tooth disease type 2: Pathomechanism insights and phenotypic spectrum widening. Eur. J. Neurol..

[26] Ding M., Shen K. (2008). The role of the ubiquitin proteasome system in synapse remodeling and neurodegenerative diseases. BioEssays.

[27] Kunimoto S., Kobayashi T., Kobayashi S., Murakami-Murofushi K. (2000). Expression of cholesteryl glucoside by heat shock in human fibroblasts. Cell Stress Chaperones.

[28] Hitraya E.G., Varga J., Jimenez S.A. (1995). Heat shock of human synovial and dermal fibroblasts induces delayed up-regulation of collagenase-gene expression. Biochem. J..

[29] Hiragami F., Motoda H., Takezawa T., Takabayashi C., Inoue S., Wakatake Y., Kano Y. (2009). Heat shock-induced three-dimensional-like proliferation of normal human fibroblasts mediated by pressed silk. Int. J. Mol. Sci..

[30] San Gil R., Ooi L., Yerbury J.J., Ecroyd H. (2017). The heat shock response in neurons and astroglia and its role in neurodegenerative diseases. Mol. Neurodegener..

