# Supplementary material for: Impaired DNAJB2 Response to Heat Shock in Fibroblasts from a Neuropathy Patient with DNAJB2/HSJ1 Mutation: Cystamine as a Potential Therapeutic Intervention
Source: Neurol Int. 2025 May 9;17(5):73. doi: 10.3390/neurolint17050073 (PMC12113656; doi:10.3390/neurolint17050073)
Supplement: Supplementary file 1 [file neurolint-17-00073-s001.zip › neurolint-3562912-supplementary.pdf]

neurology international

MDPI

Case Report

Impaired DNAJB2 response to heat shock in fibroblasts from a neuropathy patient with *DNAJB2/HSJ1* mutation: Cystamine as a potential therapeutic intervention

Raj Kumar Pradhan [1], Nikolas G. Kinney [1], Brigid K. Jensen[1] and Hristelina Ilieva [1,*]

[1] Jefferson Weinberg ALS Center, Department of Neuroscience, Thomas Jefferson University, 900 Walnut Street, JHN 4th Floor Suite 400, Philadelphia 19107, PA, United States

* Correspondence: hristelina.ilieva@jefferson.edu

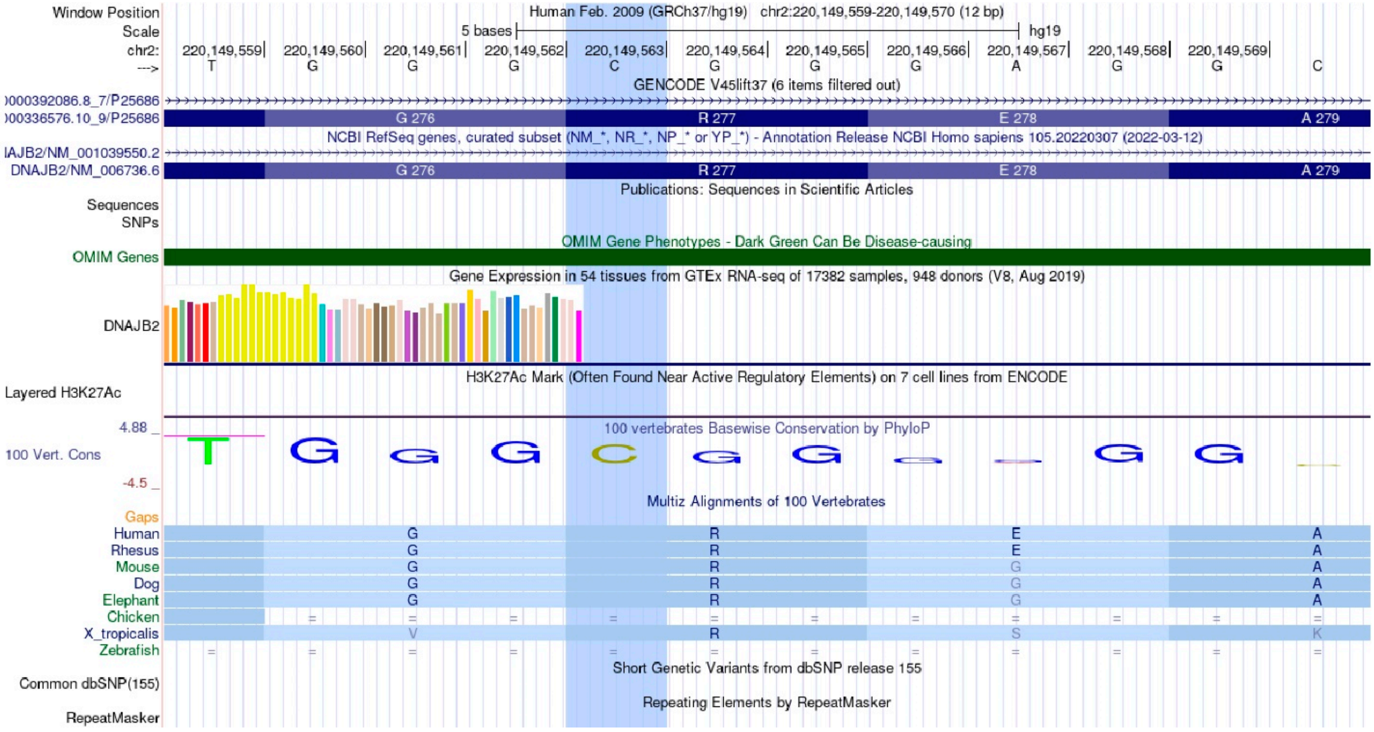

Figure S1: This figure displays the region of c.823+6C>T mutation within the *DNAJB2* gene located in chromosome 2. It also shows the region of specific nucleotide change due to the mutation. For transcript a, it is in a splice site, and algorithms predict that it will strengthen the splice site possibly causing aberrant splicing. For transcript b it falls in the coding sequence and will lead to an amino-acid change from arginine to tryptophan at position 277 of the amino acid sequence. Arginine is highly conserved among species at that site. The conservation of this site highlights the potential impact of this mutation on the gene's function. (Reference: http://genome.ucsd.edu)

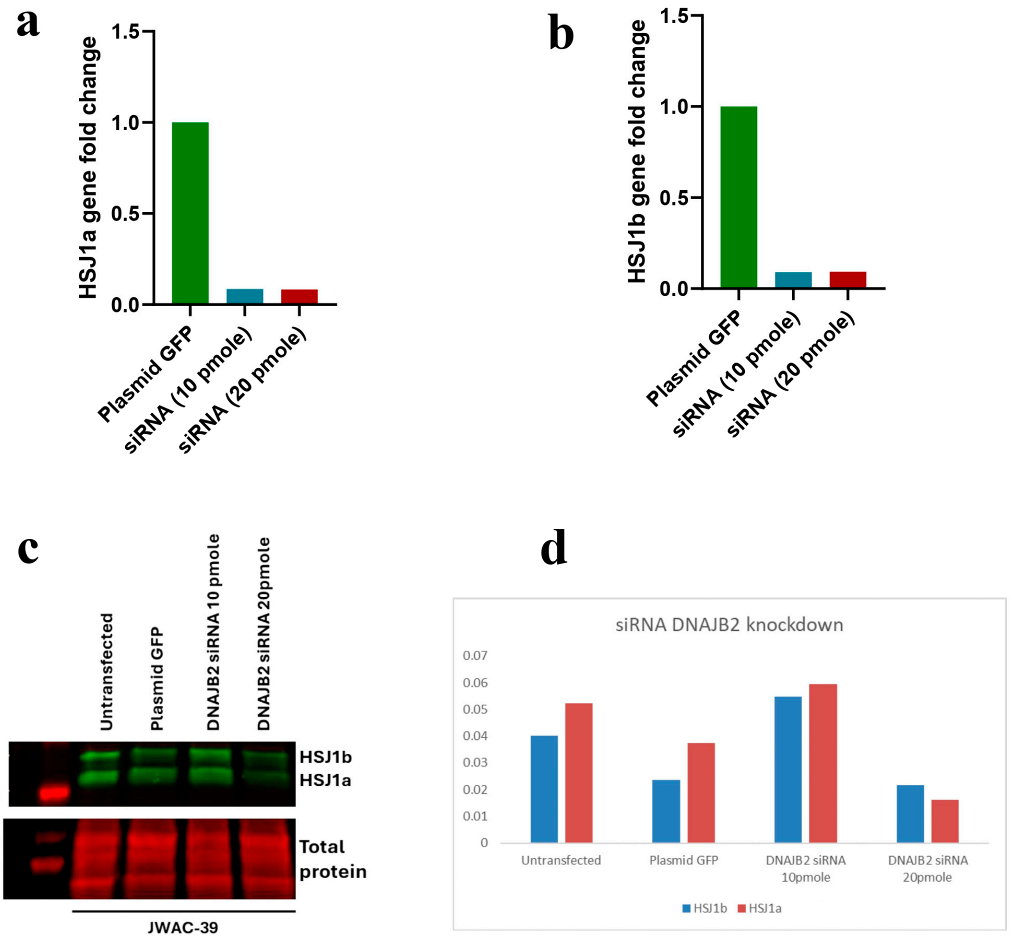

Figure S2: siRNA knockdown of DNAJB2 on the expression of DNAJB2 and HSP70. 2a. Expression of *HSJ1a* gene in the siRNA knockdown cells with different concentrations of DNAJB2 siRNA. 2b. Expression of *HSJ1b* gene in the siRNA knockdown cells with different concentrations of DNAJB2 siRNA. 2c. The upper panel is the representative immunoblot image showing the expression of HSJ1a (36kDa) and HSJ1b (40 kDa) bands in JWAC-39 cells. The lower panel represents their respective total protein stain with uniform comparable loading of proteins (20ug/lane). 2d. Densitometric analysis of HSJ1a and HSJ1b proteins expressed relative to the total protein in the patient fibroblasts (JWAC-39) (n=1) transfected with different concentrations of DNAJB2 siRNA.

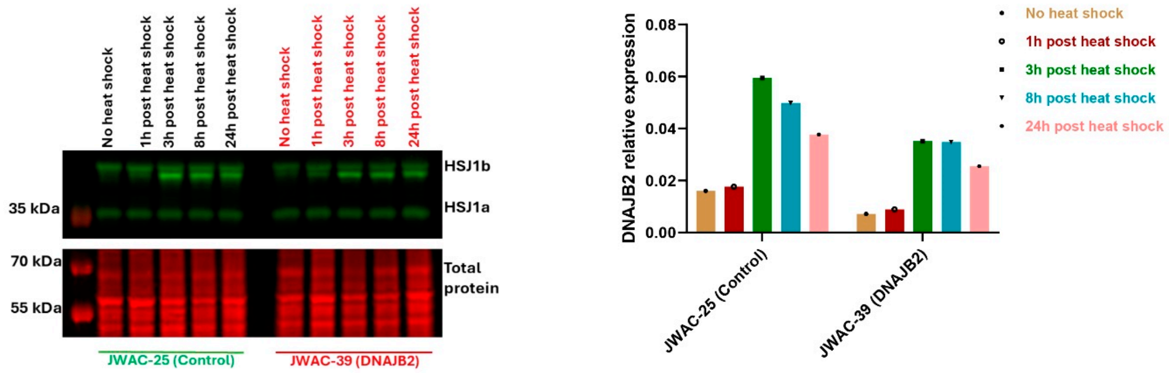

Figure S3: Expression of DNAJB2 (HSP40) at different time-points post one-hour of heat shock. 3a. The upper panel is the representative immunoblot image showing the expression of HSJ1a (36kDa) and HSJ1b (40 kDa) bands in control (JWAC-25), left and DNAJB2 (JWAC-39) fibroblasts, right, detected with anti-DNAJB2 antibody at 0, 1, 3 ,8 and 24 hours post one-hour of heat shock. The lower panel represents their respective total protein stain with uniform comparable loading of proteins (20ug/lane). 3b. Densitometric analysis of HSJ1b proteins expressed relative to the total protein in the control (JWAC-25) and patient fibroblasts (JWAC-39) (n=1)

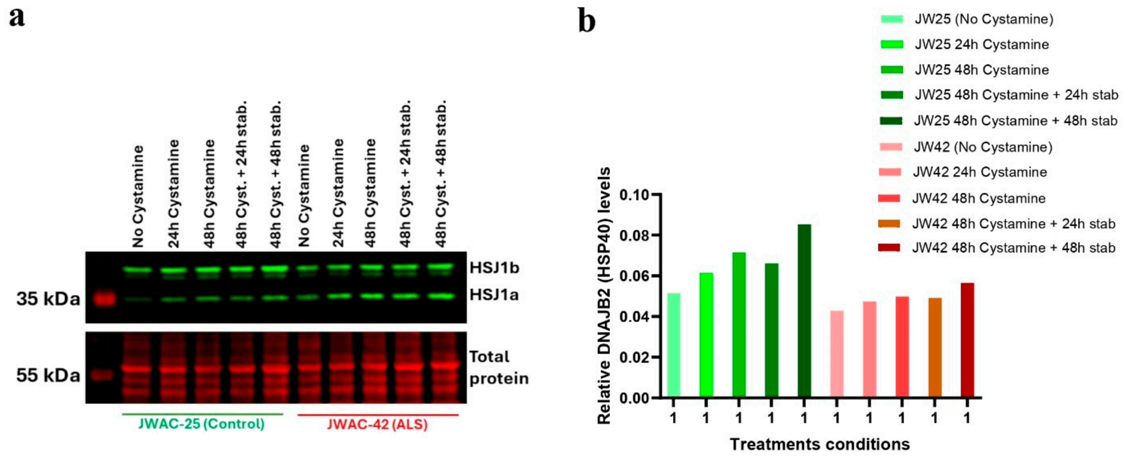

Figure S4: Effect of Cystamine pretreatment on the levels of DNAJB2 (HSP40) at different time-points. 4a. The upper panel is the representative immunoblot image showing the expression of HSJ1a (36kDa) and HSJ1b (40 kDa) bands in control (JWAC-25), left and JWAC-42 fibroblasts, right, detected with anti-DNAJB2 antibody with different exposure time and washout period of Cystamine. The lower panel represents their respective total protein stain with equal/comparable loading of proteins (20ug/lane). 4b. Densitometric analysis of HSJ1b proteins expressed relative to the total protein in the control (JWAC-25) and JWAC-42 fibroblasts (n=1)

Table S1: Primers used for different transcripts in the experiments

Gene	Forward primer	Reverse primer
Human *HSJ1a*	CTCATGCCCCTTGGACAG	AGAACACATCTGCGGGTTTC
Human *HSJ1b*	CCCAGCACCAAGATCCAG	AGAGGATGAGGCAGCGAGAG
Human *GAPDH*	GGAGCGAGATCCCTCCAAAAT	GGCTGTTGTCATACTTCTCATGG

Table S2: Primers used for Sanger Sequencing

Gene	Accession number	Forward	Reverse	Variant
Human *HSJ1a*	NM_001039550.2	CTCAGAGCTTCAGAACCGGG	CTCAGAACACATCTGCGGGT	DNAJB2:c.823+6C>T
Human *HSJ1b*	NM_006736.6	CTCAGAGCTTCAGAACCGGG	GATGGACTGCGTTTGGTTGC	DNAJB2:c.829C>T (p.Arg277Trp)
